# Supplementary material for: Are carbon emissions trading and green financial instruments synergistic? -Comprehensive quantitative research based on content analysis
Source: PLoS One. 2024 Mar 7;19(3):e0298601. doi: 10.1371/journal.pone.0298601 (PMC10919601; doi:10.1371/journal.pone.0298601)
Supplement: S1 Data — (ZIP) [file pone.0298601.s001.zip › Data/地级市Z_F工作报告.zip!/╣π╢1⁄2/╜╥╤⌠╩╨/└┤╫╘╣1⁄2╓┌║┼í╛┬φ┐╦╩2╛▌═°í┐_2012.docx]

各位代表：

　　现在，我代表市人民政府，向大会作政府工作报告，请予审议，并请政协各位委员和其他列席人员提出意见。

过去五年工作

　　过去五年，我们在省委、省政府和市委的正确领导下，在市人大及其常委会和市政协的监督支持下，深入贯彻落实科学发展观，团结带领全市人民，同舟共济化危机，攻坚克难保增长，凝心聚力促和谐，大力推进经济社会发展，较好地完成了“十一五”发展规划和市四届人大一次会议确定的目标任务。

　　——经济实力明显增强。2010年生产总值首次突破千亿元大关，预计2011年达到1230亿元，固定资产投资658.45亿元，社会消费品零售总额573.27亿元，外贸出口36.97亿美元，地方财政一般预算收入48.98亿元，五年年均分别增长17%、35.2%、27.6%、21.2%、28.9%，全部实现翻一番以上。项目建设不断突破，五年累计完成固定资产投资2064亿元；开工建设省、市重点项目585个，竣工投产422个。揭阳潮汕机场竣工通航；厦深高铁揭阳段正在推进；中委广东石化炼油、中海油粤东LNG一体化、中电投揭阳物流中心项目成功引进；惠来电厂1、2号机组建成投产，3、4号机组基本建成。发展载体不断夯实，大南海国际石化综合工业园获省批准设立，珠海（揭阳）产业转移工业园被列为省级示范园。金融支持力度不断加大，开展四批银企合作对接、525家企业获得金融机构授信500.99亿元，各项贷款余额比2006年末增长155.4%，财政连续两年共安排2000万元作为企业贷款贴息，市区和揭西农信社成功改制为农村商业银行。内外经贸不断拓展，组织企业到郑州、合肥、武汉、重庆等地举办揭阳产品展销及经贸洽谈活动，赴阿联酋、美国、荷兰等国参加产品展销活动，与162个国家地区建立了贸易关系。

　　——产业结构得到优化。工业化发展步伐加快，规模以上工业增加值年均增长34.8%，重化工业占生产总值比重五年提高12.2个百分点，三次产业结构由2006年的14.1:51.2:34.7调整为2011年的10.5：58.7：30.8。自主创新能力逐步提高，新增中国高新技术企业43家、国家级技术创新示范企业1家、省级民营科技企业54家，省级工程技术研发中心14家、省级以上企业技术中心18家，实施国家和省科技项目324项、专利授权量3352件，注册商标26795件、中国驰名商标10件、省著名商标57件、省名牌产品59个。优势特色产业改造提升，五金机械、纺织服装和精密装备制造被列为省市共建产业，荣获“中国塑料时尚鞋之都”称号，建成国家不锈钢制品质量监督检验中心，获准筹建国家纺织品服装产品质量监督检验中心（广州）揭阳服装产品分中心，揭东经济开发区被列为“国家新型工业化产业示范基地”，新增规模以上工业企业785家、限额以上商业企业1176家、上市公司2家。商贸服务产业稳步发展，金属材料城、玉都广场、国际服装城、国际商品城首期建成开业，连续成功举办5届玉器节、3届五金不锈钢制品博览会和3届普宁衬衣节。

　　——城乡面貌逐步改观。全市城镇化率达47.3%，市区建成区面积比2006年扩大1倍多。城镇建设取得新成效，编制城镇体系规划等13个重大规划和50平方公里新城区控制性详细规划，推进市区两个30亿工程和县城“五个一”、中心镇“三个一”工程建设，完成“三旧”改造项目229个9430.13亩，建成揭阳楼文化广场、念慈体育馆等一批重点市政设施，新（改）建公路2869.5公里，建成市医疗废物处理中心和9个污水处理厂，首获省卫生城市称号。农业农村工作不断加强，利用园地山坡地开发补充耕地8.05万亩，建设标准农田25.81万亩；完成水利工程投资31.8亿元；拥有农业龙头企业34家、农业标准化示范区50个、省级现代农业园区9个、农民专业合作社270个；造林6.13万公顷，森林覆盖率提高至53.2%；玉湖新农村建设经验全面推广，乡镇通村公路全部实现硬底化，村卫生所实现全覆盖，689个行政村设立文化室，793个行政村建成“雨污分流”处理工程。

　　——社会建设扎实推进。累计投入教育资金153.3亿元，新（扩）建学校905所460万平方米，新增中小学学位22万个，增配教师2.1万名，基本普及高中阶段教育。列入国家级非物质文化遗产9项、省级29项、省级文物保护单位7处，新（改、扩）建市县图书馆、文化馆、博物馆3.69万平方米，新增镇级文化站7200平方米，新建文化室590个、农家书屋692家，每万人拥有公共文化设施228平方米，潮剧《还官记》荣获最高政府奖“文华优秀剧目奖”。开展忠诚教育和感恩奉献教育。举办第三届粤东侨博会、首届玉文化节、两届特色文化节和两届揭西生态旅游文化节。新增国家4A级旅游景区2个。公办基层医疗机构100%实施基本药物制度，创建“三级甲等”医院1家。人均拥有体育场地0.96平方米。人口计生工作跃上新台阶，2011年人口自然增长率、符合政策生育率分别为5.98‰ 、97.78%。殡改成果得到巩固提高。完成对口支援汶川卧龙镇恢复重建任务。“五五”普法教育取得实效。开展社会治安十大专项整治行动，完成县镇两级综治信访维稳中心建设任务，连续三次荣获“全省综治工作优秀市”称号。没有发生重特大安全事故和产品质量、食品药品安全重大事故。物价、统计、供销、气象、地震、科协、红十字会、人民防空、人民武装、国防动员、广播电视、新闻出版、妇女儿童、民族宗教、外事侨务、对台事务、文史档案、国有资产管理等工作取得新进步。

　　——人民生活持续改善。2011年财政民生支出占公共财政总支出72.8%，比2006年提高8.4个百分点。累计新增城镇就业13.1万人，下岗再就业3.3万人，转移农村劳动力50.94万人。2011年城镇居民人均可支配收入1.69万元，农民人均纯收入6925元，年均分别增长11.3%、9.8%。城乡居民储蓄存款余额807.33亿元，年均增长13.1%。城镇居民人均住房面积28.99平方米，新增机动车14.3万辆。医疗保险覆盖率达到95%。13.46万名城乡居民享受最低生活保障待遇。建设保障性住房25万平方米，2295户城镇住房困难家庭实现“住有所居”。残疾人社会保障和服务体系不断完善。累计解决农村饮水不安全人口151.72万人；投入扶贫开发资金7.13亿元，4.25万户20.9万人实现脱贫。

　　——依法行政有效加强。定期向市人大及其常委会报告工作、向市政协通报情况，办复省、市人大代表议案、建议239件，政协委员提案、建议意见560件。完善市政府工作规则，建立市政府常务会议学法制度，充分发挥专家咨询组、经济顾问的作用，广泛听取和吸纳民主党派、工商联、无党派人士和人民团体的意见建议，重视群众来信来访，扎实推进政府科学、民主、依法决策。完成新一轮政府机构改革和第四轮行政审批制度改革，建成市、县、镇、村四级电子监察系统，实行首问责任制等12项效能制度、重点企业“绿卡”保护制度和行政首长问责制，督查问责力度不断加大，审计监督和行政监察职能得到较好发挥，行政执行力明显提升。

　　2011年是迎接新挑战、实现新突破、取得新成果的一年。我们在市委的坚强领导下，积极应对国际国内经济增长放缓、国家宏观政策收紧等因素影响，解放思想谋发展，群策群力保增长，坚定不移地走加快科学发展道路，以建设幸福新揭阳为目标，着力转变发展方式，着力提高发展质量，着力化解社会矛盾，着力解决改革发展中存在的深层次问题，有效地克服了前进中碰到的困难，有力地促进了经济与社会协调发展、质量与效益同步提升，在复杂多变的经济环境中取得了新成就，预计全年生产总值增长15%，地方财政一般预算收入增长26.7%，实现了“十二五”发展良好开局。

　　五年来的奋斗历程，让我们更加深刻地体会到:必须坚持加快发展不停步，全力推动经济持续快速增长；必须坚持转型发展不放松，着力调整优化产业结构；必须坚持统筹发展不减弱，切实加快城乡一体化进程；必须坚持共享发展不动摇，让发展成果惠及广大人民群众；必须坚持执政为民不懈怠，不断加强政府自身建设。这些既是对过去实践的经验总结，也是今后政府工作中应该把握的重要原则。

　　各位代表，五年来的发展成就振奋人心，这是省委、省政府和市委正确领导的结果，是全市人民同心同德、顽强拼搏的结果，也是社会各界大力支持、通力合作的结果。借此机会，我谨代表市人民政府，向全市各条战线的干部群众，向驻揭部队、武警官兵、人民警察和各民主党派、人民团体、各界人士，向一直以来关心支持我市改革发展的港澳台同胞、海外侨胞、外出乡亲以及国际友人，表示衷心的感谢并致以崇高的敬意！

　　在肯定成绩的同时，我们也清醒地认识到，当前我市经济社会发展仍存在不少困难和问题，突出表现在：一是经济发展水平偏低，2010年生产总值和地方财政一般预算收入分别仅占全省的2.2%、0.9%，人均分别相当于全省的38.6%、15.3%。二是产业竞争力不强，高新技术企业和民营科技企业仅占企业总数的0.8%，高新技术产品增加值仅占全市规模以上工业增加值的7.3%。三是城镇化水平较低，落后全省18.9个百分点。四是社会建设仍然滞后，每万人医生数和警察数分别仅相当于全省的76.2%、46.9%。五是人才智力支撑乏力，人才总量仅占全省的2%，高级职称人数仅占全市专业技术人才的3%。六是行政服务效能有待提高，是全省两个未建行政服务中心的地级市之一。对此，我们一定要高度重视，采取更加扎实有效的措施，在加快发展中逐步予以解决。

今后五年工作

　　今后五年，是揭阳全面实现“十二五”发展规划和“十年大发展”目标任务的攻坚阶段，也是推进揭阳科学发展的倒逼转型期。当前，欧美债务危机持续发酵，世界经济复苏曲折多变，国家宏观调控依然趋紧，全省各地竞相发展形势催人，我们既要面对加快转型升级的倒逼压力，又要面临加速发展、奋力赶超的现实挑战。展望今后五年，我们的机遇大于挑战，希望多于困难。我国仍将处于可以大有作为的重要战略机遇期，工业化、城市化和农业现代化快速推进，消费结构和产业结构升级蕴藏着巨大的需求潜力，经济持续向好的基本面没有改变，特别是国家实施《珠江三角洲地区改革发展规划纲要》和《海峡西岸经济区发展规划》、我省建设海洋经济强省和促进粤东地区加快发展的决策部署、汕潮揭一体化进程的加速推进，为打造粤东发展极提供了难得的机遇，为建设幸福新揭阳开辟了广阔的空间。我们要以只争朝夕、时不我待的精神，抢抓机遇，克服困难，努力在跨越发展的轨道上迈出新步伐，在奋发进取的征程上实现新作为。

　　新一届政府工作的总体要求是：高举中国特色社会主义伟大旗帜，以邓小平理论和“三个代表”重要思想为指导，深入贯彻落实科学发展观，认真贯彻中央、省委省政府的决策部署和市第五次党代会精神，紧紧围绕“打造粤东发展极，建设幸福新揭阳”的核心任务，大力实施“创新驱动、项目带动、文化推动、高端拉动、生态联动”发展战略，着力启动“空港经济、海港经济”两大引擎，坚持不懈地推进民生建设，努力把揭阳建设成为“重化工业基地、商贸流通之都、人文日新古邑、岭南特色水城、粤东上善新区”，赶超进位，后来居上，奋力开创科学发展新局面。

　　根据以上总体要求，我们确定新一届政府的工作理念是：强化效率、彰显公平、惠民执政、绿色发展。

　　今后五年，我们将竭力做到：

　　一心一意谋发展。加快科学发展是“打造粤东发展极，建设幸福新揭阳” 的首要任务。慢发展对不起当代，不发展耽误下一代，乱发展贻害千万代。我们要牢牢把握主题主线，紧紧围绕核心任务，坚持“好字当头、能快则快”，狠抓第一要务不放松，心无旁骛抓落实，力争一年上一个台阶，全力推动经济社会跨越发展。今后五年，我市经济增长预期目标是：生产总值年均增长16%，人均生产总值年均增长14.7%，固定资产投资年均增长25%，社会消费品零售总额年均增长24.4%，外贸出口年均增长22%，地方财政一般预算收入年均增长26%。

　　积极主动调结构。转方式调结构是“打造粤东发展极，建设幸福新揭阳”的必由之路。我们要坚决摒弃粗放式发展模式，大力推进信息化与工业化、金融资本与产业资本相融合，全力加快产业转型升级，积极构建重化工业和现代服务业“双核驱动”的现代产业体系，打造空港经济新市和海港经济大市，提升综合竞争力，努力实现速度和结构质量效益相统一、经济发展与人口资源环境相协调、消费投资出口相促进，使发展更加全面、更加协调、更加可持续。

　　坚定不移抓改革。深化改革开放是“打造粤东发展极，建设幸福新揭阳”的核心动力。历史证明，自强不息的揭阳人民从来就有敢为人先的勇气，从来就有开放包容的胸怀，从来就有奋发有为的精神。眼界绘宏图，魄力铸伟业。我们一定要毫不动摇地坚持改革方向，深入推进重点领域和关键环节的改革，敢于摆脱束缚、推倒围墙、打破框框。毫不动摇地走创新发展之路，建立健全区域创新体系，加快建设创新型城市。毫不动摇地扩大对内对外开放，自觉把揭阳发展放到全省、全国和全球总体格局中定位，以国际视野、科学精神、战略思维认识揭阳、建设揭阳、发展揭阳。

　　千方百计惠民生。增进民生福祉是“打造粤东发展极，建设幸福新揭阳”的目的归宿。坚定不移地走发展惠民之路，通过合理的制度安排维护社会公平，让惠民、利民成为政府一切工作的出发点和落脚点。我们一定要强化对人民群众的朴素感情，带着深厚感情做工作；一定要密切联系群众，深入群众，围绕人民群众的普遍性、核心性、迫切性诉求做工作；一定要牢固树立民生改善与经济发展齐头并进、幸福指数与发展指标同步提升的理念，带着惠民富民的目标做工作，积极倡导包容性增长，扎实推进惠民计划，大力实施富民工程，努力增加城乡居民收入，提高基本公共服务均等化水平，开创全民共建、共享、共富的发展新格局。

　　扎扎实实促和谐。构建和谐社会是“打造粤东发展极，建设幸福新揭阳”的根本保障。我们一定要坚持以人为本、为民执政，加强社会建设和管理创新，最大限度激发社会活力，最大限度增加和谐因素，最大限度减少不和谐因素，切切实实维护人民群众的利益不受损害，切切实实为人民群众排忧解难，切切实实化解阻碍揭阳发展的矛盾问题，实现社会和谐稳定发展。

　　我们期望，五年以后，呈现在全市人民面前的将是一个更加美好、更加幸福的新揭阳。这个新的揭阳将是：

　　——重化工业基地集聚效应明显。临港资源得到有效开发，一批重大产业项目建成发挥效益，石化产业链条不断延伸，重化产业实现集群发展，到2016年，重化工业增加值占生产总值40%左右，成为沿海新兴重化工业城市。

　　——商贸流通之都规模迅速壮大。水陆空交通枢纽优势和传统商贸优势充分发挥，专业市场繁荣活跃，电子商务快速成长，物联网技术得到推广应用，空港物流、海港物流蓬勃发展，现代物流体系基本形成，到2016年，商贸业增加值占生产总值18%左右，初步建成立足粤闽赣、辐射全国、面向世界的商贸流通基地和综合性物流中心。

　　——人文日新古邑焕发时代活力。文化创造活力持续迸发，文化事业和文化产业繁荣发展，到2016年，每万人拥有公共文化设施252平方米，文化产业增加值占生产总值8%左右，成功创建省文明城市和国家历史文化名城，形成富有潮汕特色的城市文化品牌，建成“人文精神日新、文化事业繁荣、文化产业发达、文化创新活跃”的文化名邑。

　　——岭南特色水城彰显独特魅力。三江流域生态持续改善，市区水乡风貌逐步恢复，城市建设“一年见成效、三年大改观、五年大变样”，打造天蓝、地绿、水清、城美的宜居城市，到2016年，市区建成区面积超过150平方公里，全市城镇化率达到60%左右、森林覆盖率达到55.3%以上，建成定位清晰、布局合理、功能完善的城镇群。

　　——粤东上善新区结出丰硕成果。社会事业长足进步，社会大局和谐稳定，到2016年，每万人教师数、医生数、警察数分别达到108人、15人、8人以上，城镇居民人均可支配收入、农民人均纯收入年均分别增长11.8%、13.6%，人人享有更高水平的社会保障，生活更加殷实、更有尊严、更加幸福。

2012年重点工作

　　2012年是新一届政府工作的起始之年，也是揭阳建市20周年。做好今年工作意义重大，影响深远，人民寄予厚望。我们要深入贯彻中央经济工作会议、省委十届十一次全会和市第五次党代会精神，更加注重扩大内需，更加注重改革创新，更加注重优化结构，更加注重改善民生，更加注重维护稳定，努力推动经济社会更好更快发展，为新一届政府工作开好局、起好步。全年主要预期目标是：生产总值增长16%，单位生产总值能耗下降3.66%，主要污染物排放量控制在省下达指标内，规模以上工业增加值增长31.5%，固定资产投资增长30%，社会消费品零售总额增长24%，外贸出口增长21%，地方财政一般预算收入增长27.5%，居民消费价格涨幅控制在4%左右，城镇登记失业率控制在3.5%以内，人口自然增长率控制在7‰以内，城镇居民人均可支配收入增长11.8%，农民人均纯收入增长13.1%。

　　新的一年，我们将着力抓好以下十方面工作：

　　（一）拉动经济较快增长

　　大力推进重点项目建设。突出抓好111个年度重点项目建设，完成计划投资314.36亿元。同时开工建设中委广东石化炼油、中海油粤东LNG一体化、中电投揭阳物流中心等大项目，形成撬动揭阳跨越发展的重要支点。实现惠来电厂3、4号机组核准投产。抓好康美药业优质特色饮片、青岛啤酒年产60万千升啤酒、广东领航年产150台铣床、五洲龙新能源汽车环保电池等工业项目建设。完成电网建设投资8亿元，确保78万千伏安主变容量及91.2千米输电线路建成投产。

　　大力开展招商引资。统筹全市招商引资工作，完善招商分配激励机制，对各县（市、区）引入而落户异地的项目，实行税收合理分成。抓好大南海国际石化综合工业园、珠海（揭阳）产业转移工业园等园区配套建设，重点面向大型国有企业、跨国公司、国内骨干民营企业、台资企业和揭阳乡贤开展招商选资，争取引进更多对产业形成和结构优化起引领作用的旗舰型、科技型项目。深入研究国家产业发展政策，谋划和储备一批打基础、利长远的未来发展项目。

　　大力拓展消费市场。继续实施揭货“北上西进”计划，组织名优产品到大城市举办展销会。搞活商贸流通，打造区域品牌，办好第二届玉文化节、第五届五金不锈钢制品博览会和第九届普宁衬衣节，支持国际服装城申报广东商品国际采购中心，加快药品城、玉都广场（二期）等工程建设，加强电子商务平台建设，支持物流企业发展。扩大“万村千乡市场工程”覆盖面，力争农家店覆盖全市85%的乡镇。

　　大力推进对外经贸合作。鼓励企业创建出口名牌及并购国际品牌，申报自营进出口业务，支持企业拓展国际市场，重点开拓俄罗斯、东欧等新兴市场，稳定扩大外贸出口。启动一类口岸查验基础设施建设，实现揭阳港全水域对外开放。争取获批设立揭阳潮汕机场航空口岸。积极推进汕潮揭一体化，主动融入海峡西岸经济区，善于借力珠三角的辐射带动，进一步扩大对台经贸、旅游、农业等领域的合作，密切与港澳、东盟的友好往来和经贸文化交流。

　　（二）加快产业转型升级

　　启动空港经济引擎。以揭阳潮汕机场为依托，围绕登岗、砲台、地都三镇和经济开发试验区，规划建设空港经济区，积极构建以航空运输为核心、以航空物流为先导、以临空型高新技术产业和现代服务业为重点的空港产业体系，打造先进制造业的集聚区、科技创新的核心区、体制创新的先行区、对台经贸的探索区、吸引外资的密集区和集约利用土地的示范区。重点抓好234平方公里空港经济区战略规划编制工作，加速园区基础设施建设，争取一批产业项目落户动工，形成粤东经济新亮点。

　　全力发展海港经济。依托深水良港和对外开放一类口岸，充分发挥重大项目龙头带动作用，积极构建以港口为母体，以石油化工、电力能源、装备制造三大临港工业集群为支柱，以保税物流、陆上物流、海上物流、分销物流四大临港物流中心为辐射源的临港产业体系，争当全省海洋经济综合开发试验区排头兵。加快编制我市海洋经济发展规划，推进港口码头等基础设施建设，围绕石化产业链条开展招商引资，争取落户一批中下游项目，掀起大开发大建设的新高潮。

　　推动民营经济聚合发展。扎实推进自主创新和“质量强市”工作，推动纺织服装、化工塑料、生物制药、玉器加工、五金不锈钢、玩具鞋类等传统优势产业转型升级。实施民营企业聚合发展“四大计划”。一是民营企业领军人物“榕江一期”培训计划。以行业为单位，组织民营企业领军人物，开展经济社会发展态势研究、企业资本运作、品牌打造等现代企业必备技能的培训，全面提升民营经济管理者素质，增强民营经济发展核心动力。二是民营经济优势行业聚合计划。通过整合优化产业商会、行业协会，增强行业合力，共创行业创新平台，共拓行业发展路径，推动行业资源共享、产业聚合发展。三是民营企业集合信托融资计划。按照“政（府）信（托）证（券）行（业）”合作新模式，引进权威信托机构，扶持和推动民营企业以行业为单位，实施集合信托融资计划，为民营企业快速募集发展资金开辟新路径。四是民营经济提升扩张计划。优选一批示范型企业，给予科技支持、金融服务、用地保证等政策，首期培育50家上市储备企业，今年力争有5家企业进入上市辅导期、3家企业成功上市。支持民营企业与大型国企对接，围绕产业链条开展协作配套，实现延伸发展。制订出台我市普惠民营经济发展的政策文件，设立民营经济服务中心，助力民营经济跨越发展，强化民企创富、惠民、利国的作用。

　　引进培育战略性新兴产业。支持创新型企业与中国科学院等科研院所深度衔接，顺应产业发展趋势，突破自身发展瓶颈，承接科技成果转化，延伸产业链条，实现企业在战略性新兴产业发展轨道中的自我转型提升。依托海港、空港等优势，大力引进海洋生物医药、海洋生物技术、高端装备制造、太阳能光伏、新型电子信息等战略性新兴产业。抓好我市列入省现代产业500强、自主创新100强项目建设，加速战略性新兴产业规模扩张，推动经济发展转入科技引领、创新驱动的发展轨道。力争全市战略性新兴产业产值达到550亿元，使之成为发展新动力。

　　促进县域经济健康发展。指导各县（市、区）彰显优势，理清发展思路，突出发展重点，加快产业集聚发展，建设特色鲜明、富有活力的县域经济板块。按照市第五次党代会对各县（市、区）的发展定位，重点指导普宁推进服装行业信息化进程，打造国际服装网络商城；扶持榕城区、东山区强化优势产业品牌化建设；指导揭西发展生态旅游、生态工业；支持揭东、惠来分别启动空港、海港经济建设；推动普侨区与普宁、大南山侨区与惠来紧密对接，在承担产业分工中共谋发展，实现全区域均衡发展。

　　（三）实现经济有序运行

　　保持物价基本稳定。抓好统计信息化建设，加强对经济运行的监测和研判。健全基本生活必需品价格应急处置机制，保持市场物价总体水平基本稳定。推进平价商店、蔬菜大棚、冷藏设施“三项建设”，建成覆盖城乡的平价商店网络。实施临时价格补贴与价格上涨联动机制，查处恶意炒作、串通涨价、哄抬物价等不法行为，以政府“有形的手”替人民守好“钱袋子”。

　　营造公平竞争环境。推广普宁药品打假防假创新试点经验，建立健全打假治劣工作机制，加大打假整治力度，打击制假售假、侵权盗版等违法犯罪行为，倡导诚信守法经营，规范市场经济秩序。

　　发挥金融支撑作用。创建中小微企业金融服务中心，鼓励金融机构创新服务品种，支持企业发行融资债券，促进中小微企业健康发展。培育地方中小金融法人机构，力争新增村镇银行和农村商业银行各1家、小额贷款公司3家、担保机构6家。大力引进银行机构、证券公司、行业发展基金、创业投资基金、科技引导基金等入驻揭阳，共图发展。

　　强化财税收支管理。正确处理依法治税与涵养税源关系，全面清理经济户口，加强和规范工商、税务管理，深入挖掘财税增收潜力，积极做好促产培财工作，切实提高财政保障能力。加强财政预算管理，严格控制一般性支出，集中财力保重点、保民生、保稳定，切实提高财政资金使用绩效。

　　（四）狠抓“三农”工作

　　提高农业生产水平。严格落实“米袋子”和“菜篮子”行政首长负责制，毫不放松地抓好粮食生产。大规模整治土地，改造中低产田5万亩。推进农业品牌建设，开展无公害农产品、绿色食品、有机农产品和名牌农产品认证，加强农产品地理标志保护。加大农业科技创新和推广力度，提升农业科技化、装备化、规模化、生态化水平。办好农业标准化示范区，推动农业龙头企业基本实行标准化生产。扶持发展各类农民专业合作组织，提高农业组织化程度。加强神泉、靖海等标准渔港建设，提高渔业防灾抗灾能力。确保农林牧渔业总产值增长4.5%。

　　大力兴修水利。加快引韩城乡供水工程、惠来中东部供水工程、普宁白坑湖水库综合整治工程建设，抓好列入全国中小河流近期治理规划的7宗项目整治，完成42宗小型病险水库除险加固任务，开工建设惠来赤岑水闸、邦山水闸和揭东磐岭泵站、揭西棉湖贡山泵站2宗国家大型泵站改造工程，加快三洲拦河闸应急重建工程后续项目建设步伐，扎实推进5宗千里海堤、市北河桥闸建设前期工作。

　　夯实农村发展基础。积极创建名镇名村和卫生镇、卫生村，打造生态优美、功能配套的新型农村社区。加强农村垃圾无害化处理，继续抓好农村“雨污分流”处理工程建设。加快水库移民安居工程建设。新建农村公路300公里以上，实施行政村通客运班车工程。以整村推进、产业化扶贫和劳动力转移培训为重点，坚决打好新一轮扶贫攻坚仗。积极落实中央农村工作会议精神，切实维护农民土地承包经营权、宅基地使用权、集体收益分配权等合法权利，以扎实的举措强农、惠农、富农。

　　（五）加快城市化发展步伐

　　优化交通发展环境。推动厦深高铁揭阳段建设进程，建设潮州至揭阳、汕头至揭西、揭阳至惠来高速公路揭阳段和惠来沿海公路，改造省道237线揭西灰寨至普宁麒麟段、省道238线揭西河婆至普宁池尾段、省道337线惠城至葵潭段，完成国道324线普宁池尾至惠来葵潭段、省道234线榕华大桥至锣鼓石段改建工程，加快省道335线揭东霖磐立交至揭阳高新区段改造建设，增强交通优势。

　　优化城市规划建设。强化城市建设的人本理念，实现城市建设与社会建设相融合，增强城市的服务功能和创造能力，凸显潮汕特质和水乡特色。编制完善市区控制性详细规划和专项规划。落实规划刚性约束，严查违法违规建筑行为。完成进贤门大道东及环市东路工程，推进市区水利、市政BT项目建设。深入开展市容市貌、环境卫生、道路交通集中整治行动，净化美化河边、路边、屋边“三边”环境，解决市区脏乱差等突出问题。力争成功创建省园林城市。

　　优化特色城镇建设。加快“三旧”改造建设步伐。立足各县城特色定位，进一步搞好县城规划、建设和管理，新建一批市政公用项目。扩大城镇规模，增强承载能力，力争今年城镇化率提高至55%。抓好中心镇建设，大力发展工贸镇、旅游镇、文化镇、教育镇等特色城镇。

　　（六）提升生态文明水平

　　实施“绿色揭阳”工程。强化“人树一体”理念，科学规划绿化布局。深入开展人人植绿、我与绿树共成长主题实践活动，积极创建绿色家庭、绿色校园、绿色单位、绿色村庄、绿色社区，见缝插绿、沿路植绿、满山播绿、全城尽绿。今年首期投入2亿元，建设深汕、梅汕高速公路揭阳段百里生态景观林带和绿道网。

　　实施“生态创富”工程。大力发展循环经济，积极推进清洁生产。落实节能目标管理责任制，加快淘汰落后产能，提高重点耗能行业和重点企业节能降耗水平。强化土地管理共同责任，铁腕整治违法违规用地和盗采矿产资源行为。加强土地储备管理，提高资源开发效益。坚持集约节约用地，创新用地审批制度，以“亩产论英雄”，让最小的单位土地资源消耗实现产出最大化，实现土地财富到土地创富的转变。

　　实施“环保为民”工程。开展清洁空气、重点流域环境综合整治、重金属污染防治、农村环境综合整治“四大环保为民行动”，实行从严从紧的环保政策，不符合环保要求的建设项目坚决不批，违反环保法律法规的行为坚决查处，严重污染且整治达标无望的企业坚决关停，重点抓好练江、枫江流域污染减排，力争两大流域污水处理率均达到60%；完善已建污水处理厂配套管网，争取10座列入计划的污水处理厂尽快开工，抓好5个垃圾填埋场建设，不断提高城乡治污保洁水平；严格执行《揭阳市海洋功能区划》，切实加强海洋生态保护，铸就生态竞争力。

　　（七）促进文化繁荣发展

　　加强精神文明建设。办好纪念建市20周年系列活动。开展“做新时期文明揭阳人”主题实践活动，提炼打造新时期揭阳精神。加强社会公德、职业道德、家庭美德、个人品德建设。深入开展国家卫生城市、省文明城市等群众性精神文明创建活动，大兴网络文明新风，抓好未成年人和大中学生思想道德建设，塑造良好社会心态，倡导健康幸福文化。

　　加快发展文化事业。确保文化投入不低于年度财政总支出的1%，每万人拥有公共文化设施235平方米。完成市文化中心和学宫修缮工程建设，抓好榕江观音阁建设，启动丁日昌旧居修缮工程。推进广播电视村村通、文化信息资源共享、农村电影放映和农家书屋等文化惠民工程。培育文化精品，广泛开展文化志愿活动，活跃广场文化，提升群众文化水平。启动国家历史文化名城申报工作。

　　培育壮大文化产业。加快发展创意设计、数字媒体、新闻出版、广播影视、文化娱乐、体育健身等文化产业。深化公益性文化事业单位改革，推进经营性文化单位转企改制，探索推动社会力量兴办文化产业的体制机制。大力培育外向型文化出口企业，发展壮大阳美玉雕文化创意产业园，建设南方报业集团粤东总部印务中心，创建省级文化产业基地，形成文化产业新格局。

　　（八）建设人才高地

　　树立人才引领发展的观念。落实市委关于人才的战略部署，把人才作为强市之基、富民之本、发展之源，以最大的真诚关爱人才、以最科学的态度培养人才、以最优的条件引进人才、以最适合的岗位用好人才、以最好的环境留住人才，为揭阳跨越发展提供人才支撑。

　　着力培养人才。建立榕江人才发展基金，健全政府、用人单位和社会合理分担的多元化投入机制，以项目和经费资助为杠杆，激发人才的创新活力。编制重点领域人才开发目录，引导人才向优先和鼓励发展的产业领域集聚。支持办好巨轮模具、康美药业、吉荣空调、榕泰实业四家博士后科研工作站。严格把好行政单位进人关，今后空出的编制原则上通过公开统一的招考进行补充，让更多优秀人才汇聚到行政机关中来。

　　大力引进人才。面向重点院校招录选调生到基层任职，强化基层行政力量。重点围绕医疗、教育等领域批量引进优秀大学毕业生。为企业搭建招才引智平台，支持企业引进专业技术和经济管理人才。创建“博硕人才”公寓，设立常态化的“市长与高端人才交流日”制度。按照“不求所有、但求所用”的原则，推动各级政府和重点行业、企业建立专家智库，以智力资本服务揭阳发展。

　　（九）强化社会建设

　　优先发展教育事业。全面启动“教育强市”工程，创建“教育强镇”示范点。积极发展学前教育，加快市和4个县（市）特殊教育学校规划建设，确保高中毛入学率达到88%。启动与华南师范大学的战略合作。打造名师和学科带头人。办好揭阳职业技术学院，推进市技工学校南校区建设，进一步扩大职业教育和技工教育规模。

　　增强人口健康素质。深化人口计生综合改革，探索建立市、县、镇、村“四级联创”新模式，力争计生工作达到全省中等水平。推广惠来新医改经验，深化医药卫生体制改革。启动与中国医学科学院、北京协和医学院的战略合作。扩大市卫生学校办学规模，改（扩）建县级医院2家，新增达标乡镇卫生院5家，建设全科医生培训基地。组织骨干医生开展“下基层、带徒弟、强医德”活动。推进餐饮服务食品安全量化分级管理，实现行政村药品供应网和监督网全覆盖。建设市体校，开展全民健身运动。

　　加大就业保障力度。实施农村劳动力培训工程、“千企扶千村”就业工程和创业带动就业工程，新增城镇就业3.2万人，城镇失业人员再就业1.2万人，培训农村劳动力5.8万人，转移农村劳动力16.5万人。继续扩大社会保障覆盖面，力争全市基本医疗参保人数达到565万人，其中城乡居民参保人数达到540万人。推进“新农保”试点工作，力争全市参加“新农保”人数达到107万人。

　　创新社会管理。推动社会管理向服务型管理转变。筹集揭阳市社会服务专项资金，加大政府购买社会服务力度，大力培育发展行业协会、公益社团等社会组织。抓好虚拟社会建设和管理，继续推进管理重心下移，创新农村和社区管理模式，提高群众自我教育、自我管理、自我服务水平。

　　维护社会稳定。坚持从优待警、从严治警，提高公安队伍战斗力和整体素质。始终保持“打黑除恶”高压态势，狠抓治安重点整治，坚决扫除“黄赌毒”等丑恶现象，扩大“天网工程”覆盖面，加强流动人口管理服务，推动城市畅通工程上等次，打造“平安揭阳”。建设人民来访接待厅，深入开展“大排查、大接访、大调处、大防控”活动，积极预防和化解各类社会矛盾。完善应急管理机制，提高突发事件应对能力。健全安全生产责任体系，强化重点领域安全监管，坚决遏制重特大事故发生，让人民群众在平安中感受幸福。

　　切实做好气象、地震、科协、供销、残疾人、红十字会、史志档案、新闻出版、妇女儿童、民族宗教、台湾事务、外事侨务、国防动员、人民防空、人民武装和国有资产管理等工作。

　　（十）办好十件民生实事

　　在这里，我代表市人民政府向全市人民庄严承诺，今年将扎实办好10件民生实事：一是建成农村饮水安全工程22宗，再解决31.77万农村人口饮水安全问题，让全市农民喝上安全水。二是筹措资金2.45亿元，建设公共租赁住房2450套、9.8万平方米；完成4609户农村低收入家庭住房改造任务。三是扩建市人民医院，确保成功创建“三级甲等”医院；启动市传染病院建设。四是建设义务教育规范化学校166所，新（改、扩）建幼儿园154所。五是按每人月200元标准向全市百岁老人发放百岁津贴；按每人月60元标准，向享受低保、五保待遇和重点优抚对象中80岁以上老人发放高龄津贴。六是组建公安辅警队伍，切实提高街面见警率，保一方平安。七是建设乡镇（街道）文化站11个、村（社区）文化室324个、农家书屋418家，实现乡镇（街道）综合文化站、行政村（社区）文化室全覆盖。八是完成三年扶贫开发任务，确保我市自行组织帮扶的233个贫困村、2713户贫困户、13566名贫困人口脱贫。九是大力培育扶持公益社团，重点扶持百家示范社团，组织动员民众参与社会建设，打造“公益揭阳”。十是全面规划黄岐山、紫峰山、桑浦山，加快黄岐山森林公园开发建设进程，让揭阳人民拥有最健康的“市肺”。

建设人民满意政府

　　政府的一切权力来自人民，政府的一切工作必须向人民负责，受人民监督，让人民满意。我们要始终秉承强化效率、彰显公平、惠民执政、绿色发展的工作理念，始终保持对人民群众最真切、最朴素的感情，始终因为群众的痛而痛，因为群众的喜而喜，因为群众的幸福而幸福，全力以赴办好揭阳的事情，全心全意为人民谋福祉，让新一届政府工作经得起历史检验，留得在揭阳人民心中。

　　（一）建设学习型政府。把加强学习作为政府自身建设的首要任务来抓，进一步完善政府系统学习制度，市政府常务会议每季度开展一次专题学习，各部门坚持每个月安排一次集中学习。联合北京大学光华学院、省社科院等举办各种培训班、研讨班和进修班，提高公务员综合素质和业务水平，砺练一支能干事、干成事的公务员队伍。

　　（二）建设实干型政府。深入开展“治庸、治懒、治散”专项行动，强化绩效考核和行政问责，严肃整治行政不作为、慢作为、乱作为，用抓铁有痕、踏石留印的作风狠抓工作落实。对基层高看一眼、厚爱三分，推动领导力量、项目、经费向基层倾斜，形成同呼吸、共命运的强大合力；推动政府工作人员走出机关、深入基层、走进群众，问政于民、问需于民、问计于民，惠民执政。把惠民的真与假、虚与实、多与少作为评价各级政府工作绩效的标准，砺练一支真干事、干实事的公务员队伍。深化行政体制改革，全面取消市、县设立的审批事项和前置条件，建设市行政服务中心，完善县、镇行政服务体系，推进政府网上办公和并联审批，设立中小企业投拆电话，建立市长特派员制度，打造粤东审批环节最少、行政效率最高、服务质量最好、投资软环境最优地区。

　　（三）建设法治型政府。抓好“六五”普法教育，深入开展法治城市创建活动。坚持公正文明执法，严格依法依规办事，坚决维护群众合法权益，不断提高政府公信力。健全市政府议事规则，落实社情民意调查制度、公示听证制度和重大事项集体决策制度。自觉接受市人大及其常委会的法律监督和市政协的民主监督，重视民主党派、工商联、无党派人士和工青妇等人民团体意见，加大行政监察和审计监督力度，充分发挥司法监督、新闻监督和网络监督的作用，砺练一支依法办事、文明办事的公务员队伍，切实提高依法行政水平。

　　（四）建设廉洁型政府。全面落实党风廉政建设责任制，严格执行廉洁从政“8个禁止”和“52个不准”。坚持厉行节约，反对奢侈浪费，勤俭办一切事业。深化政务公开、村务公开和厂务公开。加强惩防体系建设，加大纠风工作力度，从源头预防和治理腐败，严格要求全市公务员自觉拒收“红包礼金”，对收送“红包礼金”人员一经发现一律严处，以反腐败的实际成效取信于民，砺练一支干净干事、廉洁干事的公务员队伍，让清廉鲜明写在“打造粤东发展极，建设幸福新揭阳”的旗帜上。

　　各位代表！时代赋予了我们建设新揭阳的历史使命，这是我们的荣幸，更是我们的责任！让我们紧密团结在以胡锦涛同志为总书记的党中央周围，在省委、省政府和市委的坚强领导下，万众一心，奋发进取，开拓创新，顽强拼搏，全面夺取“打造粤东发展极，建设幸福新揭阳”的伟大胜利，为创造属于揭阳人民更加绚丽美好的明天而努力奋斗!
